# Differentiating necrotizing soft tissue infections from cellulitis by soft tissue infectious fluid analysis: a pilot study

**DOI:** 10.1186/s13017-022-00404-4

**Published:** 2022-01-08

**Authors:** Kai-Hsiang Wu, Po-Han Wu, Chih-Yao Chang, Yen-Ting Kuo, Kuang-Yu Hsiao, Cheng-Ting Hsiao, Shang-Kai Hung, Chia-Peng Chang

**Affiliations:** 1 Department of Emergency Medicine, Chang Gung Memorial Hospital, No. 6, W. Sec., Jiapu Rd., Puzih City, 613 Chiayi County Taiwan; 2 Department of Nursing, Chang Gung University of Science and Technology, Chiayi Campus, No.2, Sec. W., Jiapu Rd., Puzi City, 613 Chiayi County Taiwan; 3 Department of Emergency Medicine, Chang Gung Memorial Hospital, No.5, Fuxing St., Guishan Dist., Taoyuan City, 333 Taiwan

**Keywords:** Necrotizing soft tissue infection, Soft tissue infectious fluid, Cellulitis

## Abstract

**Background:**

We conducted this study to evaluate the characteristics of the infectious fluid in soft tissue infection and investigate the utility of the biochemical tests and Gram stain smear of the infectious fluid in distinguishing necrotizing soft tissue infection (NSTI) from cellulitis.

**Methods:**

This retrospective cohort study was conducted in a tertiary care hospital in Taiwan. From April 2019 to October 2020, patients who were clinically suspected of NSTI with infectious fluid accumulation along the deep fascia and received successful ultrasound-guided aspiration were enrolled. Based on the final discharge diagnosis, the patients were divided into NSTI group, which was supported by the surgical pathology report, or cellulitis group. The *t* test method and Fisher’s exact test were used to compare the difference between two groups. The receiver–operator characteristic (ROC) curves and area under the ROC curve (AUC) were used to evaluate the discriminating ability.

**Results:**

Total twenty-five patients were enrolled, with 13 patients in NSTI group and 12 patients in cellulitis group. The statistical analysis showed lactate in fluid (AUC = 0.937) and LDH in fluid (AUC = 0.929) had outstanding discrimination. The optimal cut-off value of fluid in lactate was 69.6 mg/dL with corresponding sensitivity of 100% and specificity of 76.9%. The optimal cut-off value of fluid in LDH was 566 U/L with corresponding sensitivity of 83.3% and a specificity of 92.3%. In addition, albumin in fluid (AUC = 0.821), TP in fluid (AUC = 0.878) and pH in fluid (AUC = 0.858) also had excellent diagnostic accuracy for NSTI. The Gram stain smear revealed 50% bacteria present in NSTI group and all the following infectious fluid culture showed bacteria growth.

**Conclusions:**

The analysis of infectious fluid along the deep fascia might provide high diagnostic accuracy to differentiate NSTI from cellulitis.

**Supplementary Information:**

The online version contains supplementary material available at 10.1186/s13017-022-00404-4.

## Background

Necrotizing soft tissue infection (NSTI) is a life-threatening soft tissue infection which is characterized by rapid progressive destruction of the muscle fascia and the surrounding soft tissue [1, 2]. A delay in diagnosis and management, including a delay in administering broad-spectrum antibiotics and surgical debridement, increases mortality and morbidity (e.g., amputation) [3, 4]. Distinguishing NSTI from cellulitis on the basis of clinical symptoms and signs may be difficult initially [5]. Several diagnostic adjuncts, including laboratory tests, the Laboratory Risk Indicator for Necrotizing Fasciitis (LRINEC) scoring system, soft tissue ultrasonography, enhanced computed tomography (CT) and magnetic resonance imaging (MRI) and fascia biopsy, have been developed to accurately diagnose NSTI [4, 6]. However, early diagnosis of NSTI remains a challenge because no single examination provides both timely and accurate information for the differential diagnosis of NSTI and non-NSTI.

The fascia necrotizes and the tissue is damaged due to the rapid progressive infection from the subcutaneous tissue to the deep facia. Imaging studies have revealed obvious fat stranding and infectious fluid accumulation along the fascia layer [7, 8]. However, no previous studies have been designed to evaluate the pathological fascial fluid. This retrospective study aimed to explore the characteristics of the infectious fluid that appears during NSTI and compare these characteristics with those of the infectious fluid that appears during cellulitis.

## Methods

### Study design and participants

This retrospective cohort study was conducted at the Chiayi Chang Gung Memorial Hospital, a tertiary care hospital in Taiwan, which has approximately 80,000 emergency department (ED) patients annually and 1300 hospital beds. This study was approved by the Institutional Review Board of Chiayi Chang Gung Memorial Hospital (No.: 201900447B0C601). Adult patients who presented to ED from April 2019 to October 2020 were enrolled based on the following three inclusion criteria: (1) severe soft tissue infection of the limbs with a clinical suspicion of NSTI by emergency medicine attending physicians, (2) infectious fluid accumulation along the deep fascia at the infection site revealed through point-of-care ultrasound (POCUS), and (3) successful ultrasound-assisted or ultrasound-guided aspiration of the infectious fluid. Patients with a history of operation, chronic osteomyelitis, and chronic or recurrent soft tissue infection (e.g., NSTI, cellulitis, and soft tissue abscess) at the site of infection were excluded. Patients with skin lesions, such as tumor or deep trauma, and who previously received antibiotics were also excluded. Soft tissue infection with abscess formation such as cellulitis with pus formation and pyomyositis was excluded due to the clear diagnosis of pus-like infectious fluid.

All clinical management decisions were made by the primary responsible ED attending physician. The standard management for patients with a clinical suspicion of NSTI presenting to the ED was the administration of broad-spectrum antibiotics and emergent orthopedic surgeon consultation for surgical intervention assessment. POCUS is a diagnostic adjunct that might help in the diagnosis of NSTI; NSTI is suspected if fluid accumulation is more than 2 mm deep along the fascia [8]. Ultrasound-guided or ultrasound-assisted aspiration was performed to obtain (1) the infectious fluid culture, and (2) obtain the Gram stain smear and the biochemical tests were used as diagnostic adjuncts when if was difficult to distinguish between NSTI and severe cellulitis. The final clinical diagnosis was made by the ED physicians and surgeons. Consequently, emergent surgical debridement including fasciotomy was then performed for the NSTI patients.

### Data collection and measurement

The electronic medical charts were reviewed and variables including age, gender, comorbidities, history of seawater contact, history of dirty farm water contact, history of an animal bite, vital sign at ED triage, laboratory data of blood and infectious soft tissue fluid, ultrasound findings of the infection site, surgery records (e.g., fasciotomy or amputation), length of intensive care unit (ICU) stay and length hospital stay and survival status at hospital discharge were recorded. All laboratory blood tests were performed within 1 h after arrival to the ED, and the infectious soft tissue fluid was collected before the administration of antibiotics. The microbiology laboratory in the hospital of the current study used the Bruker MALDI Biotyper® (Bruker, Bremen, Germany) with the matrix-assisted laser desorption/ionization mass spectrometry (MALDI-TOF MS) approach for microorganism identification and the disk diffusion tests for antimicrobial susceptibility testing [9, 10]. The cefoxitin disk diffusion method was used to detect methicillin resistance in *Staphylococcus aureus* [11, 12]. All enrolled patients were divided into an NSTI group or cellulitis group according to the discharge diagnosis. The final discharge diagnosis of NSTI was confirmed on the basis of surgical pathology report. Patients who did not have pathology reports to support the diagnosis of NSTI or who did not receive surgical intervention were enrolled in the cellulitis group.

### Data analysis

All statistical analyses were performed using SPSS and R 3.6.0 software. The *t*-test method was used to compare continuous variables in the two groups. The Fisher’s exact test was used to compare differences in the two subgroups. The continuous variables were expressed as mean and standard deviation (SD). The categorical variables were expressed as frequency (percentage [%]). Statistical significance was considered when *p* value is < 0.05. Receiver–operator characteristic (ROC) curves were calculated to determine the biochemical variables’ optimal cutoff values of the biochemical variables for the diagnosis of NSTI and the area under the ROC curve (AUC) was used to assess the discrimination ability of each variable. In general, values of 0.5, 0.5–0.7, 0.7–0.8, 0.8–0.9, and > 0.9 for AUC suggest no, poor, acceptable, excellent and outstanding discriminations, respectively [13, 14].

## Results

### Patient characteristics

**Fig. 1:**
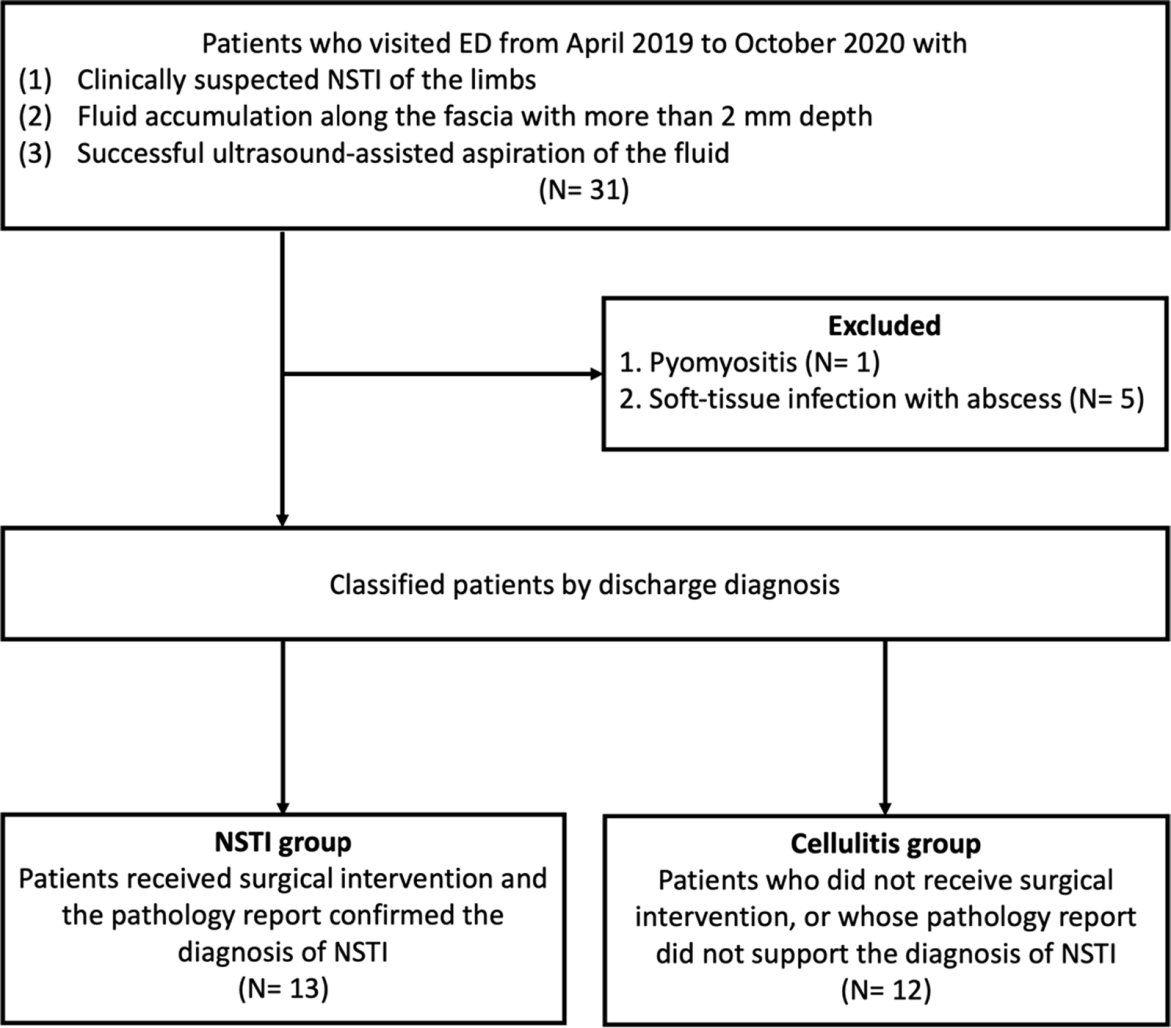
Flowchart of participants. *ED* emergency department, *NSTI* necrotizing soft tissue infection

Of the patients, 31 met the inclusion criteria (Fig. 1). Six patients were excluded due to pus-like infectious fluid found after ultrasound-guided aspiration. The final diagnosis of the six excluded patients revealed one pyomyositis and five soft tissue infections with abscess formation. Finally, 25 patients were enrolled in this study, and were categorized into the NSTI group (13 patients) and cellulitis group (12 patients). The clinical characteristics and laboratory findings on arrival to the ED were compared between the patients in the NSTI group and those in the cellulitis group. Statistical analysis of the data on continuous clinical characteristics of the two groups revealed that patients in the NSTI group had lower systolic blood pressure (SBP) and diastolic blood pressure (DBP) at the time of arrival to the ED (*p* < 0.001), whereas white blood cell count, C-reactive protein (CRP), creatinine, blood urea nitrogen (BUN), serum lactate, length of hospital stay and length of intensive care unit (ICU) stay were higher (*p* < 0.05). The results are shown in the Table 1. The analysis of the categorized clinical characteristics of the two groups revealed a higher proportion of patients with seawater or raw seafood contact in the NSTI group (*p* = 0.03), as shown in Table 2. Age, gender and proportion of comorbidities (e.g., hypertension, diabetes mellitus, liver cirrhosis, alcoholism, peripheral vascular disease and chronic kidney disease) did not differ significantly between the two groups.

**Table 1:** Comparison of continuous clinical characteristics between NSTI group and cellulitis group

Variable	NSTI			Cellulitis			*p* value
	*N*	Mean	SD	*N*	Mean	SD	
Age (years)	13	72	12.08	12	65.25	13.8	0.21
Body temperature at triage (°C)	13	36.87	1.35	12	36.76	0.76	0.81
Heart rate at triage	13	91.85	17.17	12	97.25	26.31	0.55
Respiration rate at triage	13	19.31	1.7	12	18.75	0.87	0.32
SBP at triage (mmHg)	13	105.08	29.64	12	146.58	22.6	< 0.001
DBP at triage (mmHg)	13	64.46	17.75	12	87.67	11.69	< 0.001
WBC (10^3^/uL)	13	15.98	6.55	12	9.91	3.16	0.01
Segment (%)	13	75.53	14.98	12	72.03	11.98	0.53
Hemoglobin (g/dL)	13	12.75	2.14	12	12.93	1.95	0.84
Platelet (10^3^/uL)	13	202.15	93.17	12	222.67	84.46	0.57
INR	13	1.14	0.15	8	1.03	0.07	0.08
Sodium (mEq/L)	13	134.77	3.24	12	135.33	2.74	0.64
Glucose (serum) (mg/dL)	13	181.38	91.14	11	175.73	64.93	0.87
C-reactive protein (mg/L)	11	200.36	125.79	11	71.76	63.2	0.01
Creatinine (mg/dL)	13	1.65	0.71	12	1.13	0.27	0.02
BUN (mg/dL)	11	28.66	9.95	9	14.67	6.55	< 0.001
Potassium (mEq/L)	13	3.86	0.42	12	3.72	0.45	0.41
Alanine transaminase (U/L)	13	37.38	22.12	12	37.5	27.38	0.99
Albumin (serum) (g/dL)	13	3.75	0.49	12	3.91	0.56	0.47
TP (serum) (g/dL)	13	6.43	0.74	9	6.77	0.59	0.27
LDH (serum) (U/L)	13	194.17	59.64	12	187.29	59.94	0.78
Lactate (serum) (mg/dL)	13	31.56	24.5	11	14.46	7.72	0.04
Hospital days	13	47.23	26.26	12	7.58	4.08	< 0.001
ICU days	13	1.62	2.4	11	0	0	0.03
*Infectious fluid analysis*							
Albumin (fluid) (g/dL)	13	2.19	0.96	12	1.33	0.72	0.02
LDH (fluid) (U/L)	13	5023.66	6083.34	12	315.32	307.07	0.01
Glucose (fluid) (mg/dL)	13	82.23	68.43	11	146	48.66	0.02
TP (fluid) (g/dL)	12	3.68	1.48	12	1.87	1.07	< 0.001
Lactate (fluid) (mg/dL)	13	121.98	75.63	11	24.65	13.72	< 0.001
pH (fluid)	11	8.05	0.45	8	8.6	0.32	0.01

*DBP* diastolic blood pressure, *ICU* intensive care unit, *INR* international normalized ratio, *LDH* lactate dehydrogenase, *TP* total protein, *SBP* systolic blood pressure, *SD* standard deviation, *WBC* White blood cells

**Table 2:** Comparison of categorical clinical characteristics between NSTI group and cellulitis group

Variables	NSTI		Cellulitis		Fisher's exact test *p* value
	*N*	Percentage (%)	*N*	Percentage (%)	
*Gender*					1
Male	11	84.62	10	83.33	
Female	2	15.38	2	16.67	
Wound					0.7
Yes	4	30.77	6	50.00	
No	8	61.54	6	50.00	
*Contact with seawater or raw seafood*					0.03
Yes	7	53.85	1	8.33	
No	6	46.15	11	91.67	
*Animal contact and bitten*					0.32
Yes	4	30.77	1	8.33	
No	9	69.23	11	91.67	
*Contact with farm water or dirty water*					1
Yes	4	30.77	3	25.00	
No	9	69.23	9	75.00	
*Hemorrhagic bullae*					0.24
Yes	8	61.54	4	33.33	
No	5	38.46	8	66.67	
*Serous bullae*					0.69
Yes	9	69.23	7	58.33	
No	4	30.77	5	41.67	
*Crepitus*					1
Yes	1	7.69	1	8.33	
No	12	92.31	11	91.67	
*DM*					0.41
Yes	6	46.15	4	33.33	
No	7	53.85	9	75.00	
*Chronic hepatitis*					0.65
Yes	2	15.38	3	25.00	
No	11	84.62	9	75.00	
*Chronic kidney disease*					1
Yes	1	7.69	1	8.33	
No	12	92.31	11	91.67	
*Cancer*					0.48
Yes	0	0.00	1	8.33	
No	13	100.00	11	91.67	
*Alcoholism*					0.48
Yes	0	0.00	1	8.33	
No	13	100.00	11	91.67	
*Hypertension*					0.11
Yes	5	38.46	9	75.00	
No	8	61.54	3	25.00	
*Liver cirrhosis*					0.22
Yes	0	0.00	2	16.67	
No	13	100.00	10	83.33	
*Adrenal insufficiency*					0.48
Yes	0	0.00	1	8.33	
No	13	100.00	11	91.67	
*Peripheral vascular disease*					0.48
Yes	0	0.00	1	8.33	
No	13	100.00	11	91.67	
*Amputation*					1
Yes	1	7.69	0	0.00	
No	12	92.31	12	100.00	
*Gram stain*					0.16
Bacteria	6	50.00	1	12.50	
No bacteria	6	50.00	7	87.50	

*DM* diabetes mellitus, *ICU* intensive care units

### Comparison of the infectious fluid laboratory data between the NSTI and cellulitis groups

Statistical analysis showed a significant increase in the level of lactate (*p* < 0.001), total protein (TP) (*p* < 0.001), lactate dehydrogenase (LDH) (*p* = 0.01) and albumin (*p* = 0.02) in the infectious fluid of the patients in the NSTI group as compared with that in the infectious fluid of the patients in the cellulitis group (Table 1). In addition, a significant lower glucose level (*p* = 0.02) and pH (*p* = 0.01) of the fluid were revealed in the patients in the NSTI group.

### Comparison of infectious fluid smear and culture between the NSTI and cellulitis groups

The comparison of Gram smear and fluid culture between the NSTI and cellulitis groups is shown in Table 3. The Gram stain smear of the infectious fluid showed that bacteria were present in six NSTI patients (50%) and one cellulitis patient (12.5%) (no statistically significant difference, *p* = 0.16). One NSTI patient’s smear showed polymicrobial organisms with gram-positive cocci (GPC) and gram-negative bacilli (GNB). This patient’s infectious fluid culture revealed methicillin-susceptible *S. aureus* (MSSA), *Klebsiella variicola* and *Proteus species*.

**Table 3:** Comparison of (**a**) Gram stain smear between NSTI group and cellulitis group, (**b**) infectious fluid culture between NSTI group and cellulitis group

	NSTI group *N* = 12^a^	Cellulitis group *N* = 8^a^
**(a)**		
Gram-positive coccus	3^b^	0
Gram-negative bacilli	4^b^	1
No bacteria were found	6	7
**(b)**		
*Vibrio vulnificus*	3	0
Methicillin-susceptible *Staphylococcus aureus* (MSSA)	2^c^	0
Methicillin-resistant *Staphylococcus aureus* (MRSA)	1	0
*Klebsiella Variicola*	1^c^	0
*Proteus species*	1^c^	0
No growth	6	8

^a^Two patients in NSTI group and four patients in cellulitis group were excluded due to insufficient samples for fluid culture

^b^One Gram stain smear ample in NSTI group showed both gram-positive coccus and gram-negative bacilli

^c^One infectious fluid culture showed polymicrobial infection with MSSA, *Klebsiella Variicola* and *Proteus species*

Two NSTI patients’ smears showed monomicrobial organisms with GPC and the final fluid culture showed one MSSA and one Methicillin-resistant *S. aureus* (MRSA). Three NSTI patients’ smears showed monomicrobial organisms with GNB and the culture revealed three *Vibrio vulnificus.* Only one cellulitis patient’s Gram smear presented few bacteria (< 5 bacterial cells under high power field), and the fluid culture showed no bacterial growth.

### Ability of the infectious fluid to predict the diagnosis of NSTI

ROC analysis was performed on six infectious fluid parameters that had statistically significant differences between the two groups, and the optimal cutoff values were based on the Youden index (sensitivity + specificity − 1). The predictive ability of the infectious fluid to diagnose NSTI was assessed using AUC. The AUC of lactate in fluid and LDH in fluid showed outstanding discrimination for predicting the diagnosis of NSTI. The AUC of lactate in fluid was 0.937 and displayed a sensitivity and specificity of 100% and 76.9%, respectively, at the optimal cutoff value of 69.6 mg/dL. The AUC of LDH in fluid was 0.929 and displayed a sensitivity and specificity of 83.3% and 92.3% at the optimal cutoff value of 566 U/L. Furthermore, the AUC of albumin (AUC = 0.821), pH (AUC = 0.858) and TP (AUC = 0.878) in the fluid revealed excellent discrimination for differentiating NSTI from cellulitis. The results are shown in Additional file 1: Fig. S1.

## Discussion

In this retrospective cohort study, we evaluated the characteristics of soft tissue infectious fluid above the deep fascia in comparison with the infectious fluid between NSTI patients and cellulitis patients were evaluated. In addition, the diagnostic ability of the infectious fluid to distinguish NSTI from cellulitis was evaluated. The result of current study showed that (1) LDH and lactate in the fluid had outstanding diagnostic accuracy for NSTI, (2) albumin, TP and pH of the fluid also had excellent diagnostic accuracy for NSTI, and (3) infectious fascial fluid’s cultures nay be a candidate to detect the bacterial pathogens in NSTI patients. To the best of our knowledge, this is the first study to analyze the characteristics of the infectious fascial fluid and evaluate its diagnostic ability. The infectious fluid’s laboratory tests and Gram stain smear are believed to be used as diagnostic adjuncts to help clinical physicians diagnose and manage NSTI (e.g., surgery or conservative treatment.)

NSTI is a serious infection of the deep soft tissue that causes rapid progressive destruction of the subcutaneous tissue and the deep fascia. Two distinct pathogenesis pathways have been described: (1) the evolution of NSTI with a defined portal of entry where the organisms enter the soft tissue causing local tissue infection, and (2) the evolution of NSTI without a defined portal of entry, which occurs with a nonpenetrating tissue injury (e.g., hematoma or muscle sprain) infected by transient bacteremia [1]. Once organisms reach the soft tissue, bacteria proliferate and release the endotoxins causing acute inflammation reactions including: dilatation of vessels, increased permeability of the microvasculature, emigration of the leukocytes and cytokine production by leukocytes [15]. During the acute inflammation process, exudate fluid, which contains high protein content, leaks out of the blood vessels into the soft tissues and accumulates along the deep fascia [1, 4, 8, 16]. The toxin-induced platelet–leukocyte aggregates damage the vascular endothelium and cause the capillary occlusion. Ischemic destruction of the subcutaneous tissue and deep fascia ensues as the process progresses, which results in extensive tissue necrosis and hemorrhagic bullae formation [1, 15, 17]. Cellular membranes fall apart and intracellular molecules leak, including LDH, lactate and intracellular protein, when cells die and undergo necrosis [15, 18–22]. The severity of inflammation and tissue destruction is more serious in NSTI than that in non-NSTI (e.g., cellulitis). Therefore, the current study hypothesized that the soft tissue fluid’s biochemical characteristics were different between NSTI patients and cellulitis patients. In this study, we found that several biochemical tests of the soft tissue infectious fluid might be good parameters to distinguish NSTI from cellulitis and may help physicians make timely diagnosis and management decisions.

Although no previous study has investigated the diagnostic value of the soft tissue infectious fluid, these parameters have been used as diagnostic adjuncts in other body fluids, (e.g., synovial fluid and pleural effusion) to distinguish exudate from transudate [23–29]. Several studies have revealed that lactate, LDH and pH in synovial fluid are good inflammatory markers for distinguishing septic arthritis from nonseptic arthritis [23–25]. In a retrospective observational study of 719 patients with clinically suspected septic arthritis, LDH and lactate in synovial fluid had excellent (AUC = 0.833) and acceptable (AUC = 0.760) discriminations, respectively, for predicting the diagnosis of septic arthritis [24]. In pleural effusion, pleural fluid parameters including LDH, glucose, the ratio of pleural fluid protein to serum protein, and pleural fluid pH were common diagnostic tests for discriminating between exudative and transudative pleural effusions [26–29]. This pilot study revealed the potential diagnostic value of the soft tissue infectious fluid. However, further study is required to validate the findings of the current study.

Early empirical antimicrobial therapy is one of the cornerstones of treatments for NSTI patients. The 2018 World Society of Emergency Surgery (WSES) and the Surgical Infection Society Europe (SIS-E) guidelines for the management of skin and soft-tissue infections suggested that the antibiotic treatment of NF should be aggressive (recommendation 1B), and incisional biopsy with Gram staining may be an important adjunct in early stages of suspected NSTI patients (recommendation 1C) [4]. However, the incisional biopsy with Gram staining may be difficult to perform under emergency conditions and may delay the diagnosis of NSTI. In the present study, Gram stain smear, using ultrasound-guided infectious fluid aspiration, revealed that bacteria are present in 50% of NSTI patients. Three patients in the NSTI group showed GNB in the infectious fluid’s Gram stain smear. The following fluid cultures and blood cultures all revealed the growth of *Vibrio vulnificus*. Two patients in the NSTI group showed GPC in the fluid’s Gram stain smear. The following culture revealed *S. aureus* in either blood culture or fluid culture. Therefore, we found the Gram stain smear and infectious fascial fluid culture may be optimal tests for (1) providing diagnostic evidence of infective organisms spreading through the deep fascia and (2) obtaining the culture samples before empiric antibiotics.

Although many risk indicators, laboratory parameters and diagnostic image examination, were investigated to differentiate between NSTI and cellulitis, recognizing NSTI is still challenging in the early stage of the infection [4–6, 8, 30, 31]. In this study, we found new diagnostic adjuncts that could be timely obtained in the emergency condition and may provide high discrimination for predicting the diagnosis of NSTI. However, many unanswered questions remain: Do the infectious fluid tests maintain high diagnostic accuracy in patients with chronic bilateral leg edema patients (e.g., congestive heart failure or nephrotic syndrome patients) and in patients, with previous NSTI history, encountering a new cellulitis infection in the same limb? Further studies are required to address these issues.

### Limitations

This study had three limitations that should be addressed. The first limitation was the small number of participants. It was because NSTI is a rare disease and the annual incidence of NSTI in Taiwan is about 3.26 hospitalizations per 100,000 persons/year [32]. However, the findings of this study may offer new, potentially useful diagnostic information for this rare disease. Second, this study was a retrospective design study and had its inherent limitations, such as no predesign study protocol and the possibility of unmeasured confounding. Third, this study was aimed to investigate the characteristics and the diagnostic value of the infectious fluid. Thus, the accuracy of this technique was not compared with other diagnostic scores or diagnostic examinations. Further prospective studies with a larger sample size should be conducted to validate these findings.

## Conclusions

In this retrospective cohort study, we found new timely diagnostic tests, using the infectious fluid along the fascia that might provide high diagnostic accuracy in distinguishing NSTI from cellulitis. Further research is required to validate of this study.

## Supplementary Information

**Additional file 1: Fig. S1.** Receiver operation characteristic (ROC) curves and the area under the ROC curve (AUC) demonstrating the diagnostic ability of laboratory data in infectious fascial fluid to predict the diagnosis of NF. The optimal cuff of value of each ROC curve was shown in the figure. (**a**) Albumin in fluid, (**b**) LDH in fluid, (**c**) glucose in fluid, (**d**) total protein in fluid, (**e**) lactate in fluid, and (**f**) pH of fluid.

## Data Availability

Please contact the corresponding author for data requests.

## Abbreviations

AUC: Area under the ROC curve
BUN: Blood urea nitrogen
CRP: C-reactive protein
CT: Computed tomography
DBP: Diastolic blood pressure
ED: Emergency department
GNB: Gram-negative bacilli
GPC: Gram-positive cocci
ICU: Intensive care unit
INR: International normalized ratio
LDH: Lactate dehydrogenase
MRI: Magnetic resonance image
NSTI: Necrotizing soft tissue infection
MSSA: Methicillin-susceptible *Staphylococcus aureus*
MRSA: Methicillin-resistant *Staphylococcus aureus*
POCUS: Point-of-care ultrasound
ROC: Receiver–operator characteristic
SBP: Systolic blood pressure
SD: Standard deviation
TP: Total protein
