# Supplementary material for: Differentiating necrotizing soft tissue infections from cellulitis by soft tissue infectious fluid analysis: a pilot study
Source: World J Emerg Surg. 2022 Jan 8;17:1. doi: 10.1186/s13017-022-00404-4 (PMC8742947; doi:10.1186/s13017-022-00404-4)
Supplement: Supplementary file 1 — Additional file 1: Fig. S1. Receiver operation characteristic (ROC) curves and the area under the ROC curve (AUC) demonstrating the diagnostic ability of laboratory data in infectious fascial fluid to predict the diagnosis of NF. The optimal cuff of value of each ROC curve was shown in the figure. (a) Albumin in fluid, (b) LDH in fluid, (c) glucose in fluid, (d) total protein in fluid, (e) lactate in fluid, and (f) pH of fluid. [file 13017_2022_404_MOESM1_ESM.docx]

Figure S1. Receiver operation characteristic (ROC) curves and the area under the ROC curve (AUC) demonstrating the diagnostic ability of laboratory data in infectious fascial fluid to predict the diagnosis of NF. The optimal cuff of value of each ROC curve was shown in the figure. (a) Albumin in fluid, (b) LDH in fluid, (c) glucose in fluid, (d) total protein in fluid, (e) lactate in fluid, and (f) pH of fluid.

(a) ROC curve and optimal cutoff value of albumin in fluid.

(b) ROC curve and optimal cutoff value of LDH in fluid

(c) ROC curve and optimal cutoff value of glucose in fluid

(d) ROC curve and optimal cutoff value of total protein in fluid

(e) ROC curve and optimal cutoff value of lactate in fluid

(f) ROC curve and optimal cutoff value of pH of fluid
